# Thermal plasma gasification of organic waste stream coupled with CO_2_-sorption enhanced reforming employing different sorbents for enhanced hydrogen production†

**DOI:** 10.1039/d1ra07719h

**Published:** 2022-02-21

**Authors:** Vineet Singh Sikarwar, Nageswara Rao Peela, Arun Krishna Vuppaladadiyam, Newton Libanio Ferreira, Alan Mašláni, Ritik Tomar, Michael Pohořelý, Erik Meers, Michal Jeremiáš

**Affiliations:** Institute of Plasma Physics of the Czech Academy of Sciences v. v. i., Za Slovankou 1782/3 182 00 Prague 8 Czech Republic sikarwar@ipp.cas.cz +420 703666310; Department of Power Engineering, University of Chemistry and Technology Technická 5 166 28 Prague 6 Czech Republic; Department of Green Chemistry and Technology, Ghent University 9000 Ghent Belgium; Department of Chemical Engineering, Indian Institute of Technology Guwahati North Guwahati Assam 781039 India; Department of Civil & Environmental Engineering, Hong Kong Polytechnic University 11 Yuk Choi Rd Hung Hom Hong Kong; College of Science & Engineering, James Cook University Townsville Queensland 4811 Australia; Catalytic Reaction Engineering Lab, Department of Chemical Engineering, Indian Institute of Technology Delhi Hauz Khas New Delhi India; University Center of FEI São Bernardo do Campo SP 09850-901 Brazil; ORLEN Unipetrol Centre for Research and Education (ORLEN UniCRE) Areál Chempark Litvínov-Záluží 43670 Czech Republic

## Abstract

In the past few years, rising concerns *vis-à-vis* global climate change and clean energy demand have brought worldwide attention to developing the ‘biomass/organic waste-to-energy’ concept as a zero-emission, environment-friendly and sustainable pathway to simultaneously quench the global energy thirst and process diverse biomass/organic waste streams. Bioenergy with carbon capture and storage (BECCS) can be an influential technological route to curb climate change to a significant extent by preventing CO_2_ discharge. One of the pathways to realize BECCS is *via in situ* CO_2_-sorption coupled with a thermal plasma gasification process. In this study, an equilibrium model is developed using RDF as a model compound for plasma assisted CO_2_-sorption enhanced gasification to evaluate the viability of the proposed process in producing H_2_ rich syngas. Three different classes of sorbents are investigated namely, a high temperature sorbent (CaO), an intermediate temperature sorbent (Li_4_SiO_4_) and a low temperature sorbent (MgO). The distribution of gas species, H_2_ yield, dry gas yield and LHV are deduced with the varying gasification temperature, reforming temperature, steam-to-feedstock ratio and sorbent-to-feedstock for all three sorbents. Moreover, optimal values of different process variables are predicted. Maximum H_2_ is noted to be produced at 550 °C for CaO (79 vol%), 500 °C for MgO (29 vol%) and 700 °C (55 vol%) for Li_4_SiO_4_ whereas the optimal SOR/F ratios are found to be 1.5 for CaO, 1.0 for MgO and 2.5 for Li_4_SiO_4_. The results obtained in the study are promising to employ plasma assisted CO_2_-sorption enhanced gasification as an efficacious pathway to produce clean energy and thus achieve carbon neutrality.

## Abstract 2

Bioenergy with carbon capture and storage can be an influential technological route to curb climate change to a significant extent by preventing CO_2_ discharge.

## Graphical abstract

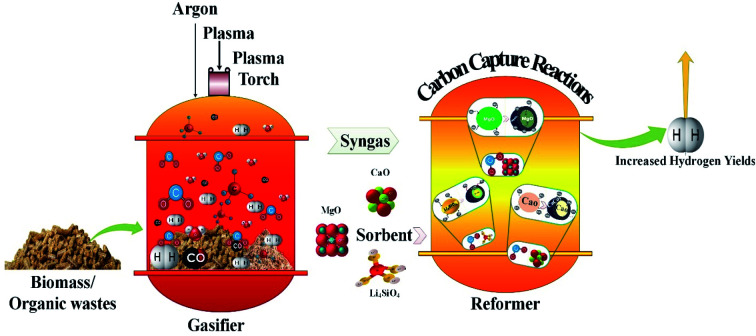

## 1. Introduction

The rising population with improving living standards have led to an increase in man-made greenhouse gas (GHG) emissions which in turn is causing global climate change. Carbon capture and storage have the potential to significantly contribute toward achieving the global warming targets set by the Intergovernmental Panel on Climate Change (IPCC)^1^ and at the Conference of the Parties (COP-21).^2^ In recent years, the elevating fears related to climate change coupled with increasing energy demand have brought the focus to developing the ‘biomass/waste-to-energy’ concept as an eco-friendly and sustainable pathway to quench global energy thirst. The characteristics of biomass such as its replenishable and carbon-neutral nature make it a lucrative energy resource.^3^ More importantly, it can be altered to a ‘carbon negative’ resource if the carbon is captured during the transformation process. This concept of coupling biomass (or organic waste)-to-energy system with carbon capture and storage (CCS) system is popularly known as BECCS (bioenergy with carbon capture and storage).^4^

It is worth noting that bioenergy can play a crucial part in curbing climate change, however, it includes grave challenges such as the efficacies of bio-based energy systems and land use practices. In the European Union (EU), the influence of bioenergy (and energy from other organic wastes) on climate has crucial significance being the largest replenishable energy source employed.^5^ It is encouraging to learn that in order to accomplish the renewable energy targets by 2020, most of the EU nations have enhanced the usage of biomass (wood). Furthermore, apart from a few solid (such as pellets) and liquid biofuels which are obtained from other countries, EU uses the bioenergy generated within its states.

It is worth noting that the philosophy of BECCS is not emphatically defined and therefore, encompasses diverse industrial and energy technologies (biomass combustion for power generation, bio-refineries, biomass transformation to liquid/gaseous fuels, *etc.*) with varying carbon dioxide discharges. In past few decades, several researchers have been working on waste/biomass gasification to generate clean energy coupled with safe and efficient waste management.^6^ The application of advanced thermal plasma technology for valorizing different organic waste streams (including biomass and hazardous waste streams) has gained attention in past years. It is on account of high temperatures and high energy fluxes gained from thermal plasma which in turn provide high destruction efficiency coupled with the ability to provide useful products such as good quality syngas, slag, *etc.*^6^ In addition, eco-friendliness and operational control are other benefits with this technology.

One of the pathways to realize BECCS is *via* CO_2_-sorption process coupled with thermal plasma gasification where *in situ* sorption of carbon dioxide takes place. It can be attained by feeding the sorbents to steam reformers^7^ or water gas shift reactors.^8^ The sorbent (such as calcium oxide, magnesium oxide, *etc.*) adsorbs CO_2_ and shifts the reaction to right hand side with higher generation of H_2_. The sorbent carbonate is then decarbonized to replenish the sorbent and to produce a concentrated CO_2_ stream.

The fundamental idea behind sorption enhanced gasification is to capture CO_2_ inside the reactor (E2, E3, E4 and E6 in Table 1) as soon as it is generated, consequently shifting the equilibrium according to Le Chatelier's principle, with an enhancement in hydrogen production.^9^ Carbon capture reactions (E10, E12, E14 in Table 1) occur at comparatively lower temperatures to adsorb CO_2_ thus forming sorbent carbonates coupled with heat release. On the other hand, at elevated temperatures, decarbonation reactions (E11, E13, E15 in Table 1) occur to recover the sorbent with concentrated CO_2_ discharge. This CO_2_ stream can be used as a feedstock for chemical synthesis or can be sent to geological storages. Several research works on CO_2_-sorption enhanced gasification have been carried out worldwide using calcium oxide,^10–13^ however, a very few have reported any other sorbent.^14–16^

**Table 1:** Important chemical reaction in carbon dioxide sorption enhanced gasification^17,18^

Equation number	Reaction name/type	Chemical equation	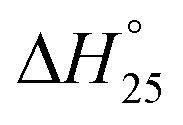 (kJ mol^−1^)
E1	Water gas-I	C + H_2_O ⇌ CO + H_2_	+131.0
E2	Water gas-II	C + 2H_2_O ⇌ CO_2_ + 2H_2_	+90.1
E3	Water gas shift	CO + H_2_O ⇌ CO_2_ + H_2_	−41.2
E4	Methane reforming	CH_4_ + 2H_2_O ⇌ CO_2_ + 4H_2_	+206.0
E5	Boudouard	C + CO_2_ ⇌ 2CO	+172.0
E6	Oxidation-I	C + O_2_ ⇌ CO_2_	−394.0
E7	Oxidation-II	2C + O_2_ ⇌ 2CO	−111.0
E8	Methanation-I	C + 2H_2_ ⇌ CH_4_	−72.8
E9	Methanation-II	2CO + 2H_2_ ⇌ CH_4_ + CO_2_	−247.0
E10	Carbonation (Ca)	CaO + CO_2_ ⇌ CaCO_3_	−178.9
E11	Decarbonation (Ca)	CaCO_3_ ⇌ CaO + CO_2_	+178.9
E12	Carbonation (Mg)	MgO + CO_2_ ⇌ MgCO_3_	−117.9
E13	Decarbonation (Mg)	MgCO_3_ ⇌ MgO + CO_2_	+117.9
E14	Carbonation (Li_4_SiO_4_)	Li_4_SiO_4_ + CO_2_ ⇌ Li_2_SiO_3_ + Li_2_CO_3_	−142.0
E15	Decarbonation (Li_4_SiO_4_)	Li_2_SiO_3_ + Li_2_CO_3_ ⇌ Li_4_SiO_4_ + CO_2_	+142.0

In the current investigation, coupling of thermal plasma gasification with CO_2_-sorption enhanced reforming in two different stages is explored from thermodynamic point of view employing three different classes of sorbents namely, calcium based sorbents (CaO) (as a model compound for high temperature sorbent), magnesium based sorbents (MgO) (as a model compound for low temperature sorbent) and alkali ceramic based sorbents (Li_4_SiO_4_) (as a model compound for intermediate temperature sorbent). Refuse derived fuel (RDF) is taken as a model compound for feedstock. The cardinal reason for the choice of model compound includes its ample availability nowadays. Moreover, its contains a lot of hydrogen which in turn is theoretically good for the production of hydrogen from the waste.

It is worth noting that plasma gasification of RDF is beneficial in comparison to conventional gasification pathways (such as fluidized bed gasification) in a way that the produced synthesis gas does contain only traces of tars (tens of mg m^−3^ ^19^ as compared to tens of g m^−3^ in fluidized bed gasification),^20^ which renders the usability of the gas for downstream enhanced gas reforming better in terms of better longevity of sorbents. The flip side of plasma gasification is the consumption of electricity for providing the energy for the allothermal process; but there is a strong possibility to adjust the power of the process to actual electricity prices in the distribution grid on account of strong flexibility of the process and therefore, it can be used as a means of transformation of surplus electricity into energy of hydrogen.^21^

**Fig. 1:**
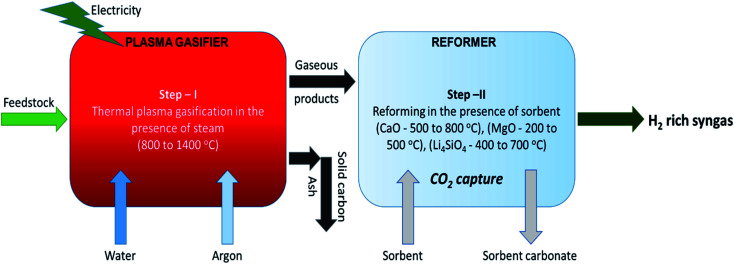
Schematic of the proposed two-steps sorption enhanced gasification with CO_2_ capture and clean energy production.

In the primary stage, thermal plasma assisted gasification of RDF takes place at higher temperatures and in the second stage, the gaseous products are reformed in the presence of sorbent at relatively low temperatures generating hydrogen rich high quality syngas coupled with *in situ* CO_2_ capture as depicted in Fig. 1. Dividing the whole process in two stages enables the individual sub-processes (plasma gasification and sorption enhanced reforming) to take place at their favorable temperatures. It is worth noting that regulating the reforming of pure gaseous products (from Step-I of the proposed process) is easier than the reforming of biomass-volatiles blend in the conventional single stage gasification.

No work to our knowledge has been published hitherto in the literature to examine the coupling of thermal plasma gasification of organic waste with sorption enhanced reforming in the presence of three different classes of sorbents for *in situ* CO_2_ capture *via* equilibrium modeling. In this study, we develop an equilibrium model for dual stage plasma assisted CO_2_-sorption enhanced gasification of RDF to evaluate the viability of proposed process in generating good quality hydrogen rich syngas. In addition, the objectives include the predictions of gas compositions, H_2_ yields, dry gas yields and LHVs as the functions of different classes of sorbents, gasification temperature, reforming temperature, steam-to-feedstock (S/F) ratio and sorbent-to-feedstock (SOR/F) ratio along with the deductions of optimal process conditions.

## 2. Methodology

A non-stoichiometric equilibrium model was developed employing the Gibbs free energy minimization approach in Aspen Plus (V11.0) thermodynamic environment using refuse derived fuel (RDF) as the model compound. The RDF used was a product from a sorting facility in Czech Republic (unrecyclable plastics mixed with unrecyclable paper and industrial wastes to meet the normalized composition). The proximate and ultimate analyses of the model compound (RDF) is given in Table 2.

**Table 2:** Proximate and ultimate analyses of the model compound (RDF)

Proximate analysis	RDF (wt%)	Ultimate analysis	RDF (wt%)
Fixed carbon	9.15	C	62.17
Volatile matter	80.24	H	8.07
Moisture	1.47	O	18.52
Ash	9.14	N	0.59
		S	0.01

The LHV (MJ Nm^−3^) of the product gas was computed based on the equation:^9^

E16 LHV = [((*m*_CO_ × 126.36) + (*m*_H_2__ × 107.98) + (*m*_CH_4__ × 358.18))/1000] MJ Nm^−3^

where, *m*_CO_, *m*_H_2__ and *m*_CH_4__ reflects the product gas components in molar percentages.

### 2.1 Assumptions in model formulation

Following assumptions were taken while developing the modeling framework: (i) all the state variables were constant inside both the reactors (gasifier and reforming reactor); (ii) the performance of the gasification reactor was not influenced by the particle size distribution of the feedstock;^13^ (iii) both the reactors were operated at isothermal conditions at 1 bar; (iv) pyrolysis took place rapidly generating hydrogen, carbon monoxide, carbon dioxide and methane as the cardinal gases;^22^ (v) pyrolysis occurred prior to the reforming reactions and char gasification (E1, E2, E5); (vi) lower heating value (LHV) were computed considering hydrogen, carbon monoxide and methane; (vii) ash was considered as a non-reactive solid; (viii) the enthalpy of formation, specific heat capacity and density were calculated using HCOALGEN and DCOALIGT property methods in Aspen Plus environment; (ix) the catalytic activities of the employed sorbents (CaO, MgO and Li_4_SiO_4_) were not considered; (x) the catalytic activity of thermal plasma was not considered.

### 2.2 Model development

The gasification of biomass/wastes entails diverse overlapping stages (for example, drying, pyrolysis, partial oxidation and gasification). Devolatization of feedstock occurs followed by heterogeneous partial oxidation of char combined with gas phase reactions and tar cracking reactions. Simplistically, tar cracking can be depicted in E17.^23^

E17 *p*C_*n*_H_*x*_ ↔ *q*C_*m*_H_*y*_ + *r*H_2_

where, C_*n*_H_*x*_ represents tar and C_*m*_H_*y*_ represents a lighter hydrocarbon *vis-à-vis* C_*n*_H_*x*_.

**Fig. 2:**
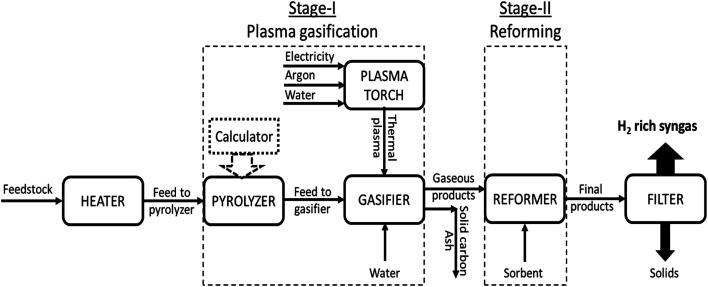
Flowsheet reflecting the simulation in Aspen Plus (V11.0).

The diverse phases which were taken into account while developing the modeling framework were the thermal plasma generation, feedstock degradation, volatile reactions and gasification, reforming, CO_2_ sorption enhancement and solid–gas separation as depicted in Fig. 2. The flow rate of model compound (RDF) was taken as 100 kg h^−1^. Three different sorbents were employed, namely, CaO, MgO and Li_4_SiO_4_. Four different cases were considered by varying gasification temperature (GT) (from 800 to 1400 °C with an interval of 100 °C), reforming temperature (RT) (from 500 to 800 °C for CaO; 200 to 500 °C for MgO and 400 to 700 °C for Li_4_SiO_4_ with an interval of 50 °C), steam-to-feedstock ratio (S/F) (from 0.8 to 2.0 with an interval of 0.2) and sorbent-to-feedstock ratio (SOR/F) (from 0.0 to 3.0 with an interval of 0.5). Each parameter was varied (keeping other variables constant) to evaluate its impact on the distribution of syngas constituents, hydrogen yield, dry gas yield and LHV and, to compute the optimal operational conditions. Water and argon were supplied to the plasma torch at constant rates of 16.8 kg h^−1^ and 3 kg h^−1^, respectively. The water was fed to the gasifier at varying rates (taking into account the water input in plasma torch) to achieve respective S/F ratio, for example, to achieve S/F of 0.8, 63.2 kg h^−1^ of water was fed to the gasifier. The sorbent was supplied to the reformer at varying rates to obtain respective SOR/F ratios.

Description of the flowsheet can be found in Table S1 of the ESI.† With reference to Fig. 2, thermal plasma was generated in the Direct Current (DC) Plasma Torch where electricity, argon and water was fed. Heater served as the plasma torch and thermal plasma input was supplied to the gasifier. The feedstock (RDF) was heated in the Heater followed by feeding to the pyrolyzer. Aspen block RYield acted as the pyrolyzer due to its capability to deduce the yields form the complex feed (non-conventional materials such as biomass, wastes, coal, *etc.*). It is assumed that the reaction (E18) took place in the pyrolyzer.^24^

E18 C_*x*_H_*y*_O_*z*_ → H_2_ + CO + CO_2_(g) + HC(g) + Tar(l) + Char(s)

where, C_*x*_H_*y*_O_*z*_ depicts the biomass/organic waste and HC reflects the hydrocarbons.

It is worth noting that a calculator block was employed to compute the yields in RYield reactor based on the equations given by other authors^22^ as depicted in Table S2 of the ESI.† These empirical relations were temperature dependent and they closely deduced the yields.

The products from pyrolyzer were fed to gasifier along with the water for steam gasification. RGibbs reactor was employed as gasifier which works on the principle of Gibbs free energy minimization to perform the phase and equilibrium calculations and compute the final yields post gasification. It was supposed that the reactions mentioned in Table 1 (E1 to E9) occurred inside the gasifier.

The gaseous products of gasifier were fed to the reformer along with the sorbent where the reforming, carbonation and decarbonation reactions took place. On the other hand, the solid products (solid carbon and ash) were separated using a Filter. Again, RGibbs reactor was used as the reformer. It is worth noting that in the absence of the loop, sorbent could not be regenerated. Therefore, fresh sorbent was continuously supplied to the reformer at varying feed rates. Finally, the products from the reformer were passed through filter to separate the solids and gases.

### 2.3 Model validation

**Fig. 3:**
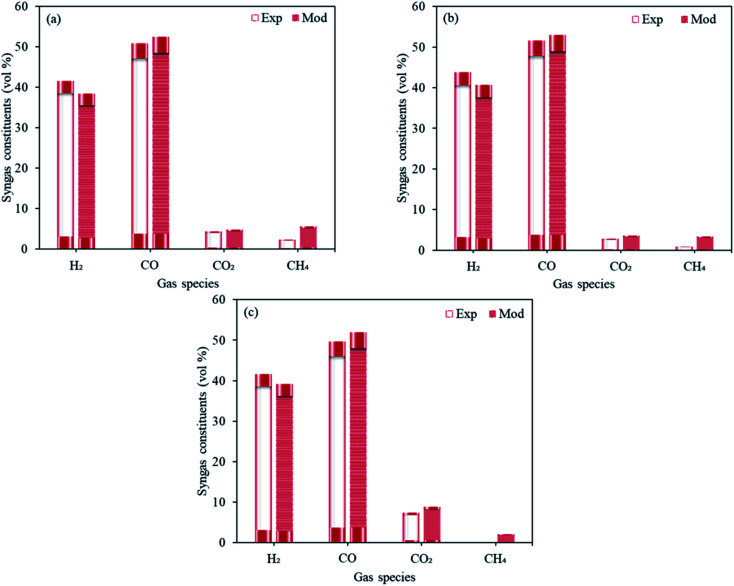
Experimental results *vs.* modeling predictions taking three different feedstocks namely, (a) sawdust, (b) pellets and (c) plastics.

The validation of the first stage of proposed model (Fig. 3) was carried out using the experimental data realized by the experimental study in our institute (Institute of Plasma Physics of the Czech Academy of Sciences).^25^ The operating conditions were taken same as that described in the study where the feed rates of sawdust, pellets (spruce wood composition with 7.4% H_2_O and 6 mm diameter) and plastics were taken as respectively 30, 30 and 11.4 kg h^−1^, in the presence of CO_2_ as the oxidizing media at 1300 °C. The dry gas fractions forecasted by the proposed model for H_2_, CO, CO_2_ and CH_4_ are in good agreement with the experimental results and the deviations are well within the reasonable limits.

No experimental study on two-stage sorption enhanced gasification of RDF with MgO and Li_4_SiO_4_ was available, so the experimental investigation by other authors^26^ using corn stalk as the feedstock in the presence of CaO for two-stage pyrolysis-gasification was chosen as a close proxy. In order to validate the mathematical model, both gasification (pyrolyzer and gasifier) and reforming temperatures were taken as 650 °C, CaO/F as 2, S/F as 1 at 1 bar pressure and the chemical composition of corn stalk was used, and resulting syngas components were compared to the experimental data. The amounts of respective syngas components (H_2_, CO, CO_2_ and CH_4_) were deduced. The proposed model predicted 88.6 vol% H_2_, 3.7 vol% CO, 1.4 vol% CO_2_ and 5.2 vol% CH_4_ whereas the experimental values were 85.1 vol% H_2_, 5.2 vol% CO, 0.1 vol% CO_2_ and 9.6 vol% CH_4_. Therefore, the modeling deductions were found to be reasonably close to the experimental data.

## 3. Predictions and discussions

### 3.1 Impact of gasification temperature

**Fig. 4:**
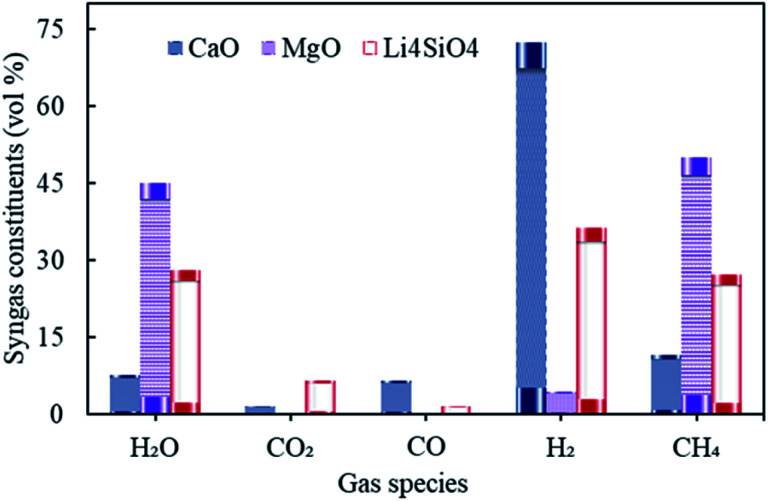
Variation in syngas constituents with variable gasification temperature (800 to 1400 °C) with CaO, MgO and Li_4_SiO_4_.

The influence of gasification temperature (in Stage-I) was assessed on syngas constituents, hydrogen yield, dry gas yield and LHV employing all three different sorbents namely, CaO, MgO and Li_4_SiO_4_ taking one at a time. The temperature of gasifier was varied from 800 to 1400 °C keeping S/F at 1 and SOR/F at 2. The reforming temperature was kept constant at 650 °C, 250 °C and 500 °C, respectively, for CaO, MgO and Li_4_SiO_4_. The choice of reforming temperatures was based on the thermodynamics of the sorption process with regard to different sorbents.^10,17,27,28^ The distribution of gaseous species is reflected in Fig. 4.

It was found that the molar fractions of syngas constituents remained the same for the entire range of gasification temperature keeping all other variables constant. As the compositions were deduced at equilibrium while keeping the reforming temperatures constant for each of the sorbent. This explained the constant distribution of syngas components with varying gasifier's temperature. It is worth noting that the gas compositions were found to be different for diverse gasification temperatures in Stage-I (as shown in Fig. S1 in the ESI†), however, after undergoing the sorption enhanced reforming in the reformer (Stage-II), the distribution of species at equilibrium became constant and thus, the effect of gasification temperature was buffered. It should be noted that this investigation was purely a thermodynamic (without kinetic considerations) study and the authors were aware that in real world scenario, some reactions would not be feasible due to kinetic limitations, mainly at the low temperatures (with MgO).

Referring to Fig. 4, hydrogen amounts employing CaO (at 650 °C) was higher with 72 vol% as compared to Li_4_SiO_4_ (at 500 °C) with 36 vol% and MgO (at 250 °C) with 4.4 vol%. On the other hand, CH_4_ was the highest (50 vol%) while MgO was employed. Reactions E1 and E2 are endothermic in nature and therefore, they were favored more at 650 °C *vis-à-vis* 500 °C and 250 °C. Also, endothermic methane reforming (E4) was strengthened in case of CaO and Li_4_SiO_4_ as compared to MgO, consuming methane and generating H_2_. This was the reason of more CH_4_ production (50 vol%) with MgO. It was deduced when CaO and Li_4_SiO_4_ were employed as sorbents, around 1.5 vol% and 6.5 vol% respectively of CO_2_ were generated on account of sorption by sorbents (E10 and E14) which in turn shifted the equilibrium for water gas shift (E3) as per Le Chatelier's principle producing more H_2_. When Mg based sorbent was used in the reformer, 0.22 vol% of CO_2_ was reported on account of adsorption by MgO (E12). Maximum H_2_O (45 vol%) and negligible CO was found in case of MgO on account of reverse water gas reactions (E1 and E2) due to lower reforming temperature of 250 °C.

As seen in Table S3 of ESI,† maximum H_2_ yield was obtained as 0.14 kg kg^−1^ of fuel for CaO and minimum as 0.0057 kg kg^−1^ of fuel for MgO. On the other hand, the dry gas yield was found to be minimum with a value of 0.53 kg kg^−1^ of fuel for syngas when MgO was employed as sorbent while it was the highest for Li_4_SiO_4_ (0.68 kg kg^−1^ of fuel). The value of LHV was maximum for MgO induced sorption enhanced reforming as compared to other employed sorbents. It was on account of higher CH_4_ concentrations in the gas which in turn contributed more to LHV calculations.

### 3.2 Impact of reforming temperature

**Fig. 5:**
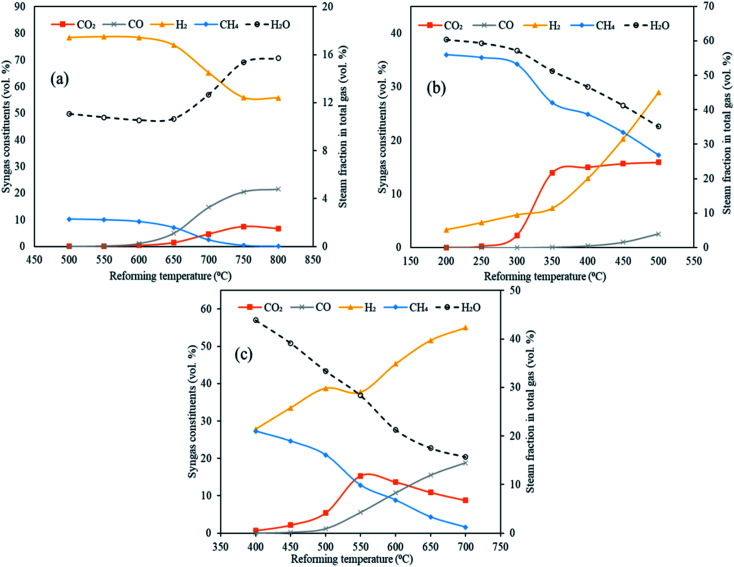
Variation in syngas constituents with variable reforming temperature employing three classes of sorbents (a) CaO sorbent (500 to 800 °C), (b) MgO sorbent (200 to 500 °C) and Li_4_SiO_4_ sorbent (400 to 700 °C). Note: *x*-axes and *y*-axes have different ranges for each set of graphs.

The distribution of syngas constituents along with carbon dioxide capture, dry gas yield and LHV were examined as a function of varying reforming temperatures (500 to 800 °C for CaO, 200 to 500 °C for MgO and 400 to 700 °C for Li_4_SiO_4_) in Stage-II, keeping the gasification temperature in Stage-I constant at 1000 °C. The sorbent-to-feedstock ratio was 2 and steam-to-feedstock ratio was 1 for the entire investigation. The alterations in gas species are depicted in Fig. 5(a)–(c) respectively, when CaO, MgO and Li_4_SiO_4_ were fed in the reformer at Step-II.

Referring to Fig. 5(a), employing CaO as sorbent in the reformer, the amounts of H_2_ decreased from 78 vol% at 500 °C to 56 vol% at 800 °C. A sharp decrease after 650 °C was noticed on account of optimal range of sorption temperature (500 to 650 °C) for CaO.^10,17^ CO_2_ was captured by CaO from 500 to 650 °C *via* carbonation reaction (E10) forming CaCO_3_. Beyond 650 °C, decarbonation began forming CO_2_ and sorbent. It was for the same reason that CO_2_ molar fraction increased beyond 650 °C from 1.5 vol% to 6.6 vol% at 800 °C. Moreover, this could be because of the steam methane reforming (E4) and water gas reaction (E2) also. On the other hand, CO continuously enhanced from 0.023 to 22 vol% as the water gas reaction (E1) was favored while CH_4_ consistently decreased as the methane reforming (E4) was strengthened, as the reforming temperature was raised. In addition, the decrease in the hydrogen and increase in the CO could possibly because of the reverse water gas shift reaction (E3).

When MgO was employed as the sorbent in the reformer, a uniform increase in H_2_ was noticed from 3.3 vol% at 200 °C to 29 vol% at 500 °C (Fig. 5(b)). This can be explained on the basis of synergistic effects of reforming reactions (E1, E2 and E4) and water gas shift (E3). However, the molar fractions of H_2_ is lower as compared to the CaO case because E1, E2 and E4 reactions are not very dominant due to comparatively lower temperatures of reformer. This also explains the higher concentrations of CH_4_ and steam fraction throughout the entire temperature range. More importantly, a clear rise in CO_2_ was seen from 300 °C due to decarbonation of MgCO_3_ (E13). Also, reactions E2 and E4 were bolstered leading to higher CO_2_ generation whereas reaction E1 was strengthened giving higher CO, with rising temperatures.

As can be seen in Fig. 5(c), a considerable and continuous rise of H_2_ was observed with rising temperature with 28 vol% at 400 °C to 55 vol% at 700 °C when lithium orthosilicate was used as a sorbent in reformer. It was on account of water gas (E1 and E2) and methane reforming (E4). This also led to decreasing CH_4_ and increasing CO amounts. A rise in CO_2_ was found from 0.66 vol% at 400 °C to 15 vol% at 550 °C as E2 and E4 reactions were favored. However, carbonation was initiated at 550 °C leading to the capture of CO_2_ by Li_4_SiO_4_ and formation of Li_2_SiO_3_ and Li_2_CO_3_.^28^ This led to a sharp decrease in CO_2_ fractions to 8.7 vol% at 700 °C.

**Fig. 6:**
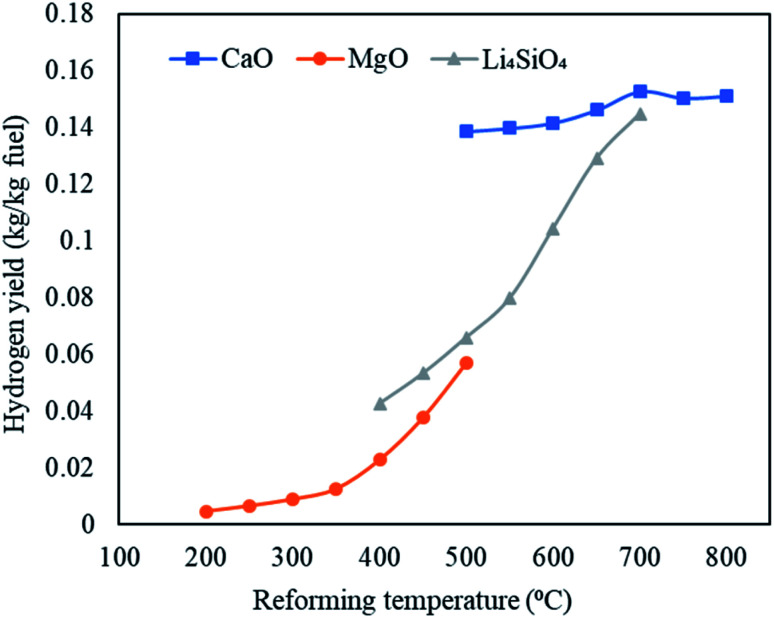
Variation in hydrogen yields (kg kg^−1^ fuel) with variable reforming temperature employing three classes of sorbents namely, CaO sorbent (500 to 800 °C), MgO sorbent (200 to 500 °C) and Li_4_SiO_4_ sorbent (400 to 700 °C).

The maximum hydrogen yields were noticed at the respective upper limits of reforming temperatures for MgO (500 °C) and Li_4_SiO_4_ (700 °C) respectively as 0.057 and 0.145 kg kg^−1^ of fuel as depicted in Fig. 6. However, for CaO, the hydrogen yield increased from 0.139 kg kg^−1^ of fuel at 500 °C to 0.153 kg kg^−1^ of fuel at 700 °C and then dropped to 0.151 kg kg^−1^ of fuel at 800 °C. Therefore, the maximum hydrogen yield with CaO was obtained at 700 °C beyond which desorption of CO_2_ began. A fairly sharp increase was seen for MgO and Li_4_SiO_4_ for their respective temperature ranges. The dry gas yield was found to increase continuously from 0.28 to 1.4 kg kg^−1^ of fuel for CaO, from 0.42 to 1.08 kg kg^−1^ of fuel for MgO and from 0.40 to 1.36 kg kg^−1^ of fuel for Li_4_SiO_4_ when the reforming temperatures were raised from 500 to 800 °C, 200 to 500 °C and 400 to 700 °C, respectively, while employing CaO, MgO and Li_4_SiO_4_ in reformer at Stage-II. When CaO was used as a sorbent, LHV decreased from 12 to 8.7 MJ Nm^−3^. Same trend was observed for MgO sorbent (13 to 9.6 MJ Nm^−3^) and Li_4_SiO_4_ sorbent (13 to 8.8 MJ Nm^−3^). The contribution of CH_4_ is maximum while deducing LHV and in all the three cases, CH_4_ amounts were decreasing leading to a drop in LHV as depicted in Table S4 of ESI.†

### 3.3 Impact of steam-to-feedstock ratio (S/F)

Steam is one of the most significant components in conventional plasma gasification and in conventional sorption enhanced gasification as it is needed for water gas (E1 and E2), methane reforming reaction (E4) and water gas shift (E3). This necessitates the exploration of steam feed rate in the gasifier in Stage-I of the current study. The total steam feed rate (steam input to plasma torch plus steam input to gasifier) was varied from 80 kg h^−1^ to 200 kg h^−1^ with an interval of 20 kg h^−1^ (S/F = 0.8 to 2.0). The other variables were constant with gasification temperature at 1000 °C, reforming temperature at 650 °C for CaO, 250 °C for MgO and 500 °C for Li_4_SiO_4_ and SOR/F at 2. The impact on the distribution of syngas components, H_2_ yield, dry gas yield and LHV were deduced as a function of varying S/F.

**Fig. 7:**
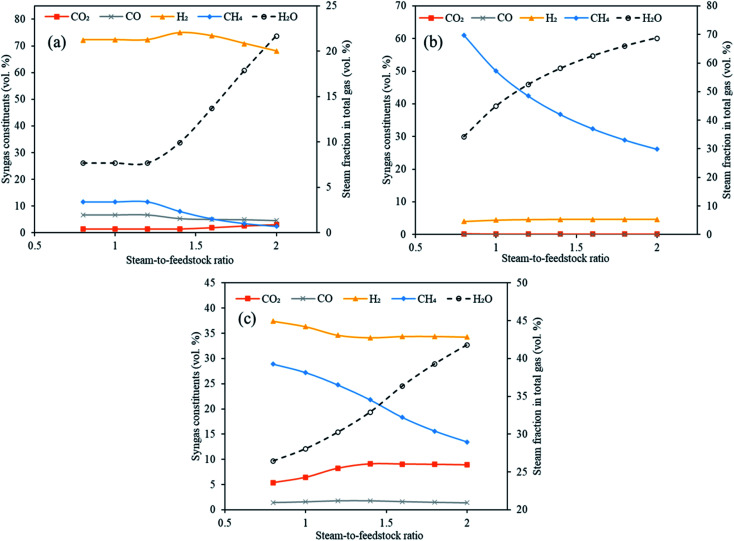
Variation in syngas constituents with variable steam-to-feedstock ratio (0.8 to 2.0) employing (a) CaO sorbent, (b) MgO sorbent and (c) Li_4_SiO_4_ sorbent. Note: *y*-axes have different ranges for each set of graphs.

Fig. 7(a)–(c) emphatically revealed the trend of carbon dioxide and other syngas distribution employing respectively CaO, MgO and Li_4_SiO_4_, when steam feed rate was raised from 80 kg h^−1^ to 200 kg h^−1^. A gradual enhancement in carbon dioxide was noticed with increasing S/F. It can be explained on the basis of alterations in partial pressure in the reaction system. As the amount of steam was increased, its partial pressure was raised leading to the shift of the water gas shift reaction (E3) towards higher production of H_2_ and CO_2_. This effect was mild when MgO was employed as sorbent and therefore, CO_2_ was found to be almost constant throughout the entire range of S/F. It should be noted that due to low temperature, the reactions of methanation (E8, E9) took place (mainly at the MgO case) because they were favored thermodynamically, however, under real conditions they are very slow at the mentioned temperatures and, therefore, could not happen to such extent without a special Ni catalyst and higher pressure and with inherent technological complications.^29^ Furthermore, it was noticed that H_2_ was first increased from 72 to 75 vol% and then decreased to 68 vol% for CaO sorbent. In the case of Li_4_SiO_4_, H_2_ concentration remained firstly decreased (from 37 to 34 vol%) and then was almost constant at 34 vol% with the increase of steam-to-feedstock ratio. On the other hand, H_2_ amounts increased mildly when MgO sorbent was fed to reformer. In the abundance of steam, methane reforming was dominant and thus produced CO_2_ and H_2_ at the cost of CH_4_ explaining a uniform reduction in CH_4_ for all three sorbents. It was seen that below certain steam-to-feedstock ratios, the variation in syngas composition was minimal on account of insufficient steam availability needed for steam reforming reactions. The change in the composition was marginal below S.F of 1.4 in the case of CaO sorbent. The alteration in H_2_, CO_2_ and CO while employing Li_4_SiO_4_ at S/F above 1.4 was found to be very mild. The deduction of trend in case of MgO was ambiguous.

It is emphatically revealed in Table S5 of ESI† that the maximum yields of H_2_ were noticed for all the three classes of sorbents namely CaO, MgO and Li_4_SiO_4_ at S/F of 2. Different trends in the dry gas yields during sorption enhanced gasification employing three different sorbents with increasing steam feeding rate (80 to 200 kg h^−1^) were noted. The highest gas yield recorded were 0.70 kg kg^−1^ of fuel, 0.53 kg kg^−1^ of fuel and 1.1 kg kg^−1^ of fuel when respectively, CaO, MgO and Li_4_SiO_4_ were fed to reformer as CO_2_ sorbents at S/F of 2. The LHV showed a decreasing trend with enhancing S/F on account of reducing methane concentrations. The highest LHV was found to be 13 MJ Nm^−3^, 22 MJ Nm^−3^ and 15 MJ Nm^−3^ at S/F of 0.8, respectively for CaO, MgO and Li_4_SiO_4_. The reason of high LHV for MgO sorbent was a greater molar fraction of CH_4_ at 0.8 S/F.

### 3.4 Impact of sorbent-to-feedstock ratio (SOR/F)

Three classes of sorbents namely, low temperature sorbent (MgO), intermediate temperature sorbent (Li_4_SiO_4_) and high temperature sorbent (CaO) were used in the Stage-II in reformer in the current investigation with an objective to capture CO_2_*in situ* and enhance H_2_ generation. The influence of sorbent feed rate (0 to 300 kg h^−1^ with an interval of 50 kg h^−1^) on H_2_ yield, carbon dioxide capture, syngas constituents, dry gas yield and LHV was examined. The gasifier temperature was constant at 1000 °C, reformer temperature at 650 °C for CaO, 250 °C for MgO and 500 °C for Li_4_SiO_4_ with S/F ratio at 1.

It is worth noting that the underlying principle of sorption enhanced gasification is sorption of CO_2_ by the employed sorbent which in turn shifts the equilibrium in water gas shift (E3) thus enhancing the H_2_ production. The sorbent after capturing the CO_2_ forms carbonate and the reactions are known as carbonation (E10, E12 and E14) with the release of energy. At elevated temperatures (different for different sorbents), decarbonation takes place (E11, E13 and E15) where sorbent is regenerated with the release of concentrated stream of CO_2_ which may be employed as a feedstock for chemical synthesis or may be sent to geological storage.

**Fig. 8:**
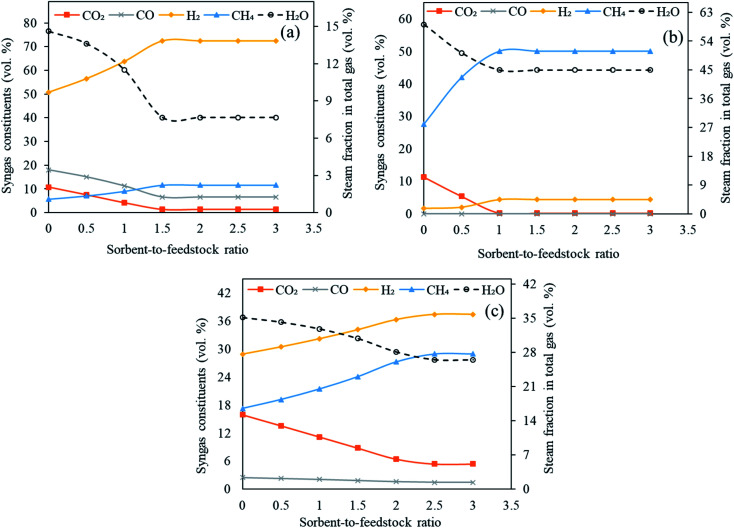
Variation in syngas constituents with variable sorbent-to-feedstock ratio (0 to 3) employing (a) CaO sorbent, (b) MgO sorbent and (c) Li_4_SiO_4_ sorbent. Note: *y*-axes have different ranges for each set of graphs.

As shown in Fig. 8(a)–(c), the molar concentrations of CO_2_ continuously decreased as SOR/F ratio was enhanced from 0.0 to 3.0. When CaO was employed, 11 vol% CO_2_ was generated at zero sorbent feeding as compared to 1.5 vol% at a SOR/F of 3. For the same pair of SOR/F, 51 vol% H_2_ was generated against 72 vol%. Same trends were noticed for MgO (11 vol% CO_2_ and 1.6 vol% H_2_ at zero sorbent feed rate as compared to 0.22 vol% CO_2_ and 4.5 vol% H_2_ at 300 kg h^−1^ sorbent feed rate) and Li_4_SiO_4_ (16 vol% CO_2_ and 29 vol% H_2_ at zero sorbent feeding as compared to 5.4 vol% CO_2_ and 37 vol% H_2_ at 300 kg h^−1^ sorbent feeding) sorbents. It was on account of the equilibrium shift in accordance with Le Chatelier's principle due to CO_2_ adsorption. CO and H_2_O were continuously reacting in water gas shift (E3) forming CO_2_ and H_2_ which explains a decreasing trend in CO and steam fraction for all the three sorbents. In addition, it was noticed that above certain sorbent-to-feedstock ratio, the changes in the composition almost ceased. The alteration in the composition was found to be marginal above SOR/F of 1.5 in the case of CaO, above SOR/F of 1 in the case of MgO and above SOR/F of 2.5 in the case of Li_4_SiO_4_ which defined the respective optimal sorbent-to-feedstock ratios. This was probably due to the availability of excess sorbent than what was needed for the adsorption reactions.

As evident from Table S6 of ESI,† the maximum hydrogen yield while CaO was employed as a sorbent was noted as 0.14 kg kg^−1^ of fuel from SOR/F of 1.5 to 3.0. In case of MgO, H_2_ yield increased slightly from 0.0027 kg kg^−1^ of fuel from SOR/F of zero to 0.0057 kg kg^−1^ of fuel at SOR/F of 1.0 and then remained constant till SOR/F of 3.0. On the other hand, Li based sorbent showed highest hydrogen yield (0.060 kg kg^−1^ of fuel) at SOR/F of 2.5 and 3.0. A decreasing trend of dry gas yields with increasing SOR/F was found. When CaO was used as a sorbent, a gas yield of 1.5 kg kg^−1^ of fuel was observed at zero SOR/F whereas 0.54 kg kg^−1^ of fuel was found at a SOR/F of 3. Similarly, as MgO and Li_4_SiO_4_ feeding were enhanced, a decline in gas yields was noticed. It was on account of CO_2_ adsorption with rising sorbent feeding rate which resulted in a decrease in dry gas yield. The highest LHV were seen at SOR/F of 3 for all the three sorbents (13 MJ Nm^−3^ with CaO, 18 MJ Nm^−3^ with MgO and 15 MJ Nm^−3^ with Li_4_SiO_4_). It was due to the improved concentrations of H_2_ and CH_4_.

## 4. Conclusions

In this investigation, an equilibrium modeling framework was developed using Aspen Plus (V11.0) thermodynamic environment with an objective to assess the feasibility of thermal plasma gasification coupled with CO_2_-sorption enhanced gasification of organic waste stream (employing RDF as model compound) using three different classes of sorbents namely, high temperature sorbents (CaO), intermediate sorbents (Li_4_SiO_4_) and low temperature sorbents (MgO) as a pathway to generate clean energy in the form of hydrogen rich syngas, capture CO_2_*in situ* and thus, achieve carbon neutrality or negativity. The impact of gasification temperature (at Stage-I), reforming temperature (at Stage-II), steam feed rate and sorbent feed rate on hydrogen yield, dry gas yield, LHV and distribution of syngas species were evaluated and optimal operational conditions were deduced. Many unit blocks were combined and temperature dependent empirical equations were used to increase the accuracy in predictions. The modeling results were validated against the published experimental studies and they both were in good agreement. This can be considered as a model which may be employed for the use any organic waste stream/biomass using allothermal steam gasification at temperatures higher than 1000 °C in the first stage.

In the proposed plasma assisted sorption enhanced gasification, the optimal CO_2_ sorption temperature was found to be 500 °C for CaO, 200 °C for MgO and 400 °C for Li_4_SiO_4_ with CO_2_ molar fractions of respectively 0.017, 0.012 and 0.669 vol%. Maximum H_2_ was noted to be produced at 550 °C for CaO (79 vol%), 500 °C for MgO (29 vol%) and 700 °C (55 vol%) for Li_4_SiO_4_. Optimal SOR/F ratio was found to be 1.5 for CaO, 1.0 for MgO and 2.5 for Li_4_SiO_4_. On the other hand, optimal S/F ratio with respect to H_2_ production were reported to be 1.4, 1.6 and 0.8 for respectively, CaO, MgO and Li_4_SiO_4_.

Future work: This study was completely a thermodynamic study. The modeling forecasts and experimental data were noted to be in good agreement qualitatively. However, in real world scenario, some reactions would not be feasible due to kinetic limitations, mostly at the lower temperatures (especially when employing MgO as a sorbent) and therefore, some amendments are necessary to improve the model predictions. The improvement in modeling deductions can be achieved by considering the reaction kinetics in the process. The aforementioned investigation is currently in progress within our lab (Lab for Plasma Chemical Technologies) and the results will soon be communicated.

## Conflicts of interest

There are no conflicts to declare.

## Supplementary Material

RA-012-D1RA07719H-s001
