# Supplementary material for: Thermal plasma gasification of organic waste stream coupled with CO2-sorption enhanced reforming employing different sorbents for enhanced hydrogen production
Source: RSC Adv. 2022 Feb 21;12(10):6122–32. doi: 10.1039/d1ra07719h (PMC8981593; doi:10.1039/d1ra07719h)
Supplement: RA-012-D1RA07719H-s001 [file RA-012-D1RA07719H-s001.pdf]

Electronic Supplementary Material (ESI) for RSC Advances.
This journal is © The Royal Society of Chemistry 2022

Thermal plasma gasification of organic waste stream coupled with CO_2-sorption enhanced reforming employing different sorbents for enhanced hydrogen production

Vineet Singh Sikarwar [a,b,c*], *Nageswara Rao Peela* [d], *Arun Krishna Vuppaladadiyam* [e,f,g], *Newton Libanio Ferreira* [h], *Alan Maslani* [a], *Ritik Tomar* [b,i], *Michael Pohořelý* [b], *Erik Meers* [c], *Michal Jeremiáš* [a]

[a] Institute of Plasma Physics of the Czech Academy of Sciences, v. v. i., Za Slovankou 1782/3, 182 00 Prague 8, Czech Republic.

[b] Department of Power Engineering, University of Chemistry and Technology, Technická 5, 166 28 Prague 6, Czech Republic.

[c] Department of Green Chemistry and Technology, Ghent University, 9000 Ghent, Belgium.

[d] Department of Chemical Engineering, Indian Institute of Technology, Guwahati, Assam -781039, India.

[e] Department of Civil & Environmental Engineering, Hong Kong Polytechnic University, 11 Yuk Choi Rd, Hung Hom, Hong Kong.

[f] College of Science & Engineering, James Cook University, Townsville, Queensland, 4811, Australia.

[g] Catalytic Reaction Engineering Lab, Department of Chemical Engineering, Indian Institute of Technology Delhi, Hauz Khas, New Delhi, India.

[h] University Center of FEI, São Bernardo do Campo, SP, 09850-901, Brazil

[i] ORLEN Unipetrol Centre for Research and Education (ORLEN UniCRE), Areál Chempark, Litvínov-Záluží 43670, Czech Republic.

Corresponding Author's Email Address: sikarwar@ipp.cas.cz; Phone# +420 703666310 (V.S. Sikarwar)

Supplementary Information

Index:

Table S1. Components of Aspen Plus flowsheet

Table S2. Empirical correlations to calculate H_2, CO, CO_2 and CH_4 yields during pyrolysis

Table S3. Hydrogen yield, dry gas yield and LHV of fuel gas employing CaO, MgO and Li_4SiO_4 with variable gasification temperature.

Table S4. Hydrogen yield, dry gas yield and LHV of fuel gas employing CaO, MgO and Li_4SiO_4 with variable reforming temperature.

Table S5. Hydrogen yield, dry gas yield and LHV of fuel gas employing CaO, MgO and Li_4SiO_4 with variable steam-to-feedstock ratio.

Table S6. Hydrogen yield, dry gas yield and LHV of fuel gas employing CaO, MgO and Li_4SiO_4 with variable sorbent-to-feedstock ratio.

Fig. S1. Variation in syngas constituents after gasification (Stage-I) with variable gasification temperature (800 to 1400 ^oC)

Table S1. Components of Aspen Plus flowsheet.

**Block / stream employed**	**Type**	**Objective**	**Basic condition (blocks)**
Feedstock	Stream	RDF feed	-
Argon	Stream	Feed to plasma torch	-
Water	Stream	Feed to plasma torch	-
Plasma	Stream	Thermal plasma feed to gasifier	-
Feed to pyrolyzer	Stream	Feed from heater to pyrolyzer	-
Water	Stream	Feed to gasifier (Stage-I)	-
Feed to gasifier	Stream	Products of pyrolyzer and feed to gasifier	-
Gaseous products of gasifier	Stream	Gaseous products from gasifier and feed to reformer (Stage-II)	-
Solid carbon and ash	Stream	Output from gasifier	-
Sorbent	Stream	Sorbent feed to reformer	-
Final products	Stream	Product gases (primarily H_2, CO, CO_2 and CH_4), solids and ash from reformer to Filter	-
Syngas	Stream	Gaseous species in final products	-
Solids / ash	Stream	Solid species in final products	-
HEATER	Block	Preheat the feed to pyrolyzer	T = 800 to 1400 ^oC; P = 1 bar
PLASMA TORCH	Block	Heater serving as DC water/Ar hybrid plasma torch	T = 800 to 1400 ^oC; P = 1 bar
PYROLYZER	Block	RYield reactor for pyrolysis (breaks down the feed into respective components)	T = 800 to 1400 ^oC; P = 1 bar
Calculator	Block	Calculator block calculates the respective yields of syngas constituents based on the temperature dependent empirical equations as mentioned in Table S2	-

GASIFIER	Block	RGibbs reactor for gasification (based on the principle of Gibbs free energy minimization)	T = 800 to 1400 ^{o}C; P = 1 bar
FILTER	Block	SSplit to separate gaseous and solid products coming from gasifier	-
REFORMER	Block	RGibbs reactor for CO_2-sorption enhanced reforming reactions (based on the principle of Gibbs free energy minimization)	T = 500 to 800 ^{o}C (CaO); T = 200 to 500 ^{o}C (MgO); T = 400 to 700 ^{o}C (Li_4SiO_4); P = 1 bar
FILTER	Block	SSplit to separate product gases and solids coming from reformer	-

Table S2. Empirical correlations to calculate H_2, CO, CO_2 and CH_4 yields during pyrolysis.

Gas (vol %)	Empirical equation
Hydrogen	$0.04694T_p - 16.96286$
Carbon monoxide	$0.0371T_p + 19.961$
Carbon dioxide	$0.000143(T_p)^2 - 0.27808T_p + 139.948$
Methane	$- 9\times10^{-5}(T_p)^2 + 0.1221T_p - 25.206$

* T_p = Pyrolysis temperature in degree Celsius

Table S3. Hydrogen yield, dry gas yield and LHV of fuel gas employing CaO, MgO and Li_4SiO_4 with variable gasification temperature.

**Variable/Sorbent**	**CaO**	**MgO**	**Li_4SiO_4**
**H_2 Yield (kg/kg of fuel)**	0.14	0.0057	0.059
**Dry gas Yield (kg/kg of fuel)**	0.54	0.53	0.68
**LHV (MJ/Nm^3)**	13	18	14

Table S4. Hydrogen yield, dry gas yield and LHV of fuel gas employing CaO, MgO and Li_4SiO_4 with variable reforming temperature.

**Variable/Sorbent**	**CaO**							**MgO**							**Li_4SiO_4**						
**Temperature (°C)**	**500**	**550**	**600**	**650**	**700**	**750**	**800**	**200**	**250**	**300**	**350**	**400**	**450**	**500**	**400**	**450**	**500**	**550**	**600**	**650**	**700**
**H_2 Yield (kg/kg of fuel)**	0.139	0.139	0.142	0.146	0.153	0.150	0.151	0.0047	0.0067	0.0090	0.013	0.023	0.037	0.057	0.043	0.053	0.066	0.079	0.104	0.129	0.145
**Dry gas Yield (kg/kg of fuel)**	0.28	0.28	0.32	0.45	0.92	1.4	1.4	0.42	0.42	0.48	0.91	0.97	1.02	1.08	0.40	0.45	0.58	1.17	1.29	1.34	1.36
**LHV (MJ/Nm³)**	12	12	12	11	9.8	8.8	8.7	13	13	12	11	10	10	9.6	13	12	11	9.3	9.4	9.1	8.8

Table S5. Hydrogen yield, dry gas yield and LHV of fuel gas employing CaO, MgO and Li_4SiO_4 with variable steam-to-feedstock ratio.

**Variable/Sorbent**	**CaO**							**MgO**							**Li_4SiO_4**						
**S/F**	**0.8**	**1.0**	**1.2**	**1.4**	**1.6**	**1.8**	**2.0**	**0.8**	**1.0**	**1.2**	**1.4**	**1.6**	**1.8**	**2.0**	**0.8**	**1.0**	**1.2**	**1.4**	**1.6**	**1.8**	**2.0**
**H_2 Yield (kg/kg of fuel)**	0.12	0.14	0.15	0.17	0.19	0.21	0.22	0.0043	0.0057	0.0069	0.0081	0.0092	0.0103	0.0127	0.053	0.059	0.065	0.074	0.084	0.093	0.101
**Dry gas Yield (kg/kg of fuel)**	0.48	0.54	0.61	0.57	0.61	0.66	0.70	0.53	0.53	0.53	0.53	0.53	0.53	0.53	0.57	0.68	0.83	0.93	0.97	1.0	1.1
**LHV (MJ/Nm³)**	13	13	13	12	10	9.5	9.0	22	18	16	14	12	11	9.8	15	14	13	12	10	9.5	8.7

Table S6. Hydrogen yield, dry gas yield and LHV of fuel gas employing CaO, MgO and Li_4SiO_4 with variable sorbent-to-feedstock ratio.

**Variable/Sorbent**	**CaO**							**MgO**							**Li_4SiO_4**						
**SOR/F**	**0.0**	**0.5**	**1.0**	**1.5**	**2.0**	**2.5**	**3.0**	**0.0**	**0.5**	**1.0**	**1.5**	**2.0**	**2.5**	**3.0**	**0.0**	**0.5**	**1.0**	**1.5**	**2.0**	**2.5**	**3.0**
**H_2 Yield (kg/kg of fuel)**	0.12	0.13	0.13	0.14	0.14	0.14	0.14	0.0027	0.0029	0.0057	0.0057	0.0057	0.0057	0.0057	0.057	0.057	0.058	0.059	0.059	0.060	0.060
**Dry gas Yield (kg/kg of fuel)**	1.5	1.1	0.80	0.54	0.54	0.54	0.54	0.78	0.65	0.53	0.53	0.53	0.53	0.53	1.1	0.97	0.86	0.76	0.68	0.65	0.65
**LHV (MJ/Nm³)**	9.8	11	12	13	13	13	13	10	15	18	18	18	18	18	9.6	10	11	13	14	15	15

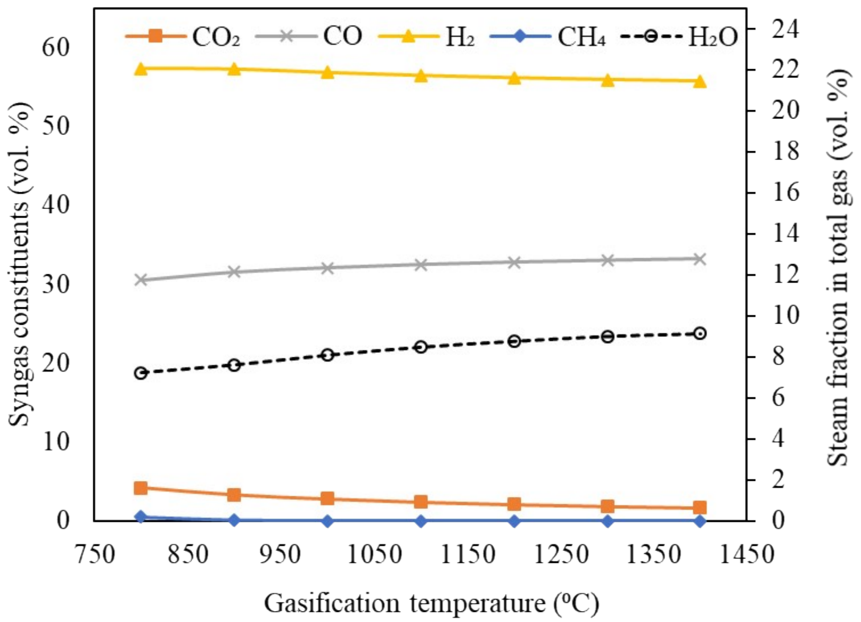

Fig. S1. Variation in syngas constituents after gasification (Stage-I) with variable gasification temperature (800 to 1400 °C)
